# R2R3-MYB Transcription Factors Regulate Anthocyanin Biosynthesis in Grapevine Vegetative Tissues

**DOI:** 10.3389/fpls.2020.00527

**Published:** 2020-05-07

**Authors:** Sha Xie, Yujuan Lei, Huawei Chen, Junnan Li, Huangzhao Chen, Zhenwen Zhang

**Affiliations:** ^1^College of Enology, Northwest A&F University, Xianyang, China; ^2^College of Food Science and Technology, Hebei Normal University of Science & Technology, Qinhuangdao, China; ^3^College of Food and Biological Engineering, Henan University of Animal Husbandry and Economy, Zhengzhou, China; ^4^Shaanxi Engineering Research Center for Viti-Viniculture, Xianyang, China

**Keywords:** anthocyanin, color pigmentation, vegetative tissues, grapevine, repressor, transcription factor

## Abstract

Anthocyanins with important physiological functions mainly accumulate in grape berry, but teinturier grape cultivars can accumulate anthocyanins in both reproductive and vegetative tissues. The molecular regulatory mechanisms of anthocyanin biosynthesis in grapevine reproductive and vegetative tissues are different. Therefore, teinturier grapevine cultivar provides opportunities to investigate transcriptional regulation of vegetative anthocyanins, and to compare with mechanisms that regulate grape berry anthocyanins. Yan73 is a teinturier *Vitis vinifera* variety with vegetative tissues able to accumulate anthocyanins, but the anthocyanin pattern and the molecular mechanism regulating anthocyanin biosynthesis in these tissues remain uncharacterized. We analyzed the anthocyanin metabolic and transcriptome profiles of the vegetative tissues of Yan73 and its male parent with HPLC-ESI-MS/MS and RNA-sequencing technologies. Yan73 vegetative tissues had relatively high 3′-OH, acylated, and methoxylated anthocyanins. Furthermore, peonidin-3-*O*-(*trans*-6-coumaryl)-glucoside is the most abundant anthocyanin in Yan73 grapevine vegetative tissues. A total of 30,17 and 10 anthocyanin biosynthesis genes showed up-regulated expression in Yan73 leaf, stem and tendril, respectively, indicating anthocyanin biosynthesis in Yan73 vegetative tissues is regulated by transcription factors. The up-regulated expression of *VvMYBA1* on chromosome 2 and *VvMYBA5*, *VvMYBA6*, and *VvMYBA7* on chromosome 14 are responsible for the anthocyanin patterns of Yan73 vegetative tissues. The expression of a set of R2R3-MYB C2 repressor genes is activated and may negatively regulate anthocyanin biosynthesis in Yan73 vegetative tissues. These findings enhance our understanding of anthocyanin biosynthesis in grapevine.

## Introduction

Anthocyanins are a class of flavonoids that determine the characteristic colors of plant fruits, flowers, and vegetative tissues. They are produced in plants in response to developmental and environmental signals and perform important physiological functions related to pollination and seed distribution (Albert et al., 2015), resistance to environmental stresses (e.g., high light irradiance, nutrient deficiency, low temperatures, and pathogen infections), and senescence (Gutha et al., 2010). Anthocyanins in reproductive tissues (e.g., berries) attract considerable attention due to their antioxidant capacity, cardiovascular disease protectiveness and antitumoral properties (Butelli et al., 2008). However, anthocyanins produced in vegetative tissues received much less attention, although they are reported to have adaptive advantages for plants. These anthocyanins in vegetative tissues often appear transiently at specific developmental stages and may be induced by environmental factors to help plants to resist environmental stresses (Chalker-Scott, 1999; Fang et al., 2018). In most grapevine, anthocyanins mainly accumulate in reproductive tissues (e.g., berries). However, in teinturier grape cultivars, anthocyanins can accumulate in both reproductive and vegetative tissues (e.g., stems, leaves, and tendrils). Indeed, in some grape varieties, anthocyanins are usually induced in vegetative tissues in response to physiological and environmental fluctuations, such as infections by the grapevine leafroll-associated virus (GLRaV) (Gutha et al., 2010) and *Botrytis cinereal* (Blanco-Ulate et al., 2015), exposure to environmental stresses [e.g., UV-B radiation (Sunitha et al., 2019)], early in plant development and autumnal senescence (Matus et al., 2017). These indicate anthocyanins are not only nutritional compounds but also important for plant in the adaptation to physiological and environmental fluctuations. This may partly explain why teinturier grapevines are resistant to mildew, powdery mildew, and phylloxera (Santiago et al., 2008). Anthocyanins were high in young teinturier grapevine vegetative tissues, decreased in mature vegetative tissues, and increased in senescing vegetative tissues, indicating anthocyanins accumulated in teinturier grapevine vegetative tissues may have an important role in protecting plants against external stresses as young or senescing vegetative tissue were more susceptible to stress damage (Guan et al., 2012). Until now, investigations of teinturier grapevine varieties have mainly focused on the anthocyanin in the berry skin and flesh (Falginella et al., 2012; Xie et al., 2018, 2019). However, the vegetative anthocyanins in teinturier grapevines have not been well studied, although this investigation can advance our understanding of anthocyanin in grapevine.

Anthocyanin accumulation in plant tissues depends on the coordinated expression of anthocyanin biosynthesis genes in the flavonoid branch of the phenylpropanoid pathway. This coordinated expression is usually controlled by a ternary MYB-bHLH-WD40 (MBW) transcription complex. In this complex, the R2R3-MYB transcription factor determines the spatio-temporal patterns of anthocyanin production in plants (Albert et al., 2014; Costantini et al., 2015). The members of the R2R3-MYB gene families encode diverse domains, which result in these genes typically mediating different anthocyanin patterns (Albert et al., 2011, 2015). Recent studies revealed that anthocyanin patterns in plants are coordinately regulated by R2R3-MYB transcriptional activators and repressors, such as DPL and PHZ activators and the PhMYB27 repressor in petunia (Albert et al., 2011, 2014), the PpMYB10.1 activator and PpMYB18 repressor in peach (Zhou et al., 2019), the CsRuby1 activator and CsMYB3 repressor in citrus (Huang et al., 2019), and MYB134 and MYB115 activators as well as MYB165 and MYB194 repressors in poplar (Ma et al., 2018). Furthermore, this transcriptional network regulating anthocyanin biosynthesis is conserved in eudicots (Albert et al., 2014). Grapevine is an important model for understanding this transcriptional regulation due to the expansion and diversification of genetic factors controlling anthocyanin biosynthesis (Matus et al., 2017). Grapevine studies have primarily focused on the *MYBA1* and *MYBA2* transcriptional activator genes within the berry color locus on chromosome 2 because they determine the variability of anthocyanin accumulation in berry skin (Walker et al., 2007). The recently identified *MYBA5*, *MYBA6*, and *MYBA7* transcriptional activator genes within the vegetative color locus on chromosome 14 control the anthocyanin biosynthesis in grapevine vegetative tissues, indicating anthocyanin accumulation in grapevine reproductive and vegetative tissues is possibly regulated by different mechanisms (Matus et al., 2017). However, this earlier study on vegetative anthocyanin mainly focused on the non-teinturier *V. vinifera* cv. ‘Pinot Noir’ young leaves and tendrils and the ‘Corvina Veronese’ buds. Much more research is needed to exactly understand the transcriptional regulation of vegetative anthocyanins in other grapevine varieties. Teinturier grapevine cultivar with high anthocyanin accumulated in their vegetative tissues provides opportunities to investigate transcriptional regulation of vegetative anthocyanins, and to compare with mechanisms that regulate grape berry anthocyanins. In addition, recent studies indicated that a set of R2R3-MYB C2 transcriptional repressors, including MYBC2-L1 (Cavallini et al., 2015), MYBC2-L2 (Zhu et al., 2019), MYBC2-L3 (Munoz et al., 2019) and MYB4-like (Perez-Diaz et al., 2016), negatively regulate anthocyanin biosynthesis in grapevine, suggesting the transcriptional activators and repressors may cooperatively regulate anthocyanin biosynthesis in grapevine; however, relatively little is known about the underlying mechanism. We recently revealed that the MYBA1 transcriptional activator and MYBC2-L1 repressor coordinately regulate anthocyanin biosynthesis of grape berry flesh (Xie et al., 2019), but it is unknown if a similar mechanism exists for grapevine vegetative tissue anthocyanin. In fact, the transcriptional repressors associated with anthocyanin biosynthesis in vegetative tissues have yet to be identified in grapevine.

**FIGURE 1:**
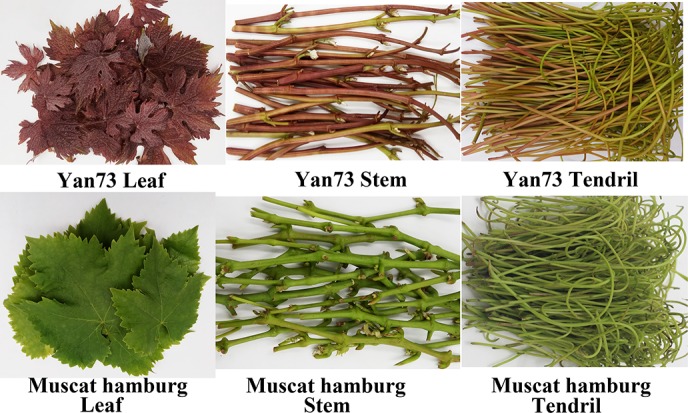
Photos of leaf, stem, and tendril tissues from Yan73 grapevine and its male parent (Muscat Hamburg).

Yan73 is a teinturier grapevine variety with anthocyanin accumulation in its vegetative tissues (e.g., leaves, stems, and tendrils) (Figure 1). The red-pigmented phenotype of Yan73 vegetative tissue is inherited from its female parent, Alicante Bouschet (Guan et al., 2012), whereas Muscat Hamburg (male parent) have green vegetative tissues. However, the anthocyanin pattern and molecular mechanism regulating anthocyanin biosynthesis in Yan73 vegetative tissues have not been elucidated. In this study, we analyzed the anthocyanin metabolic and transcriptome profiles of the leaves, stems, and tendrils of Yan73 and its male parent with HPLC-ESI-MS/MS and RNA-sequencing technologies. We then screened the RNA sequencing (RNA-seq) data to identify the differentially expressed R2R3-MYB family genes in the leaves, stems, and tendrils among Yan73 and its male parent. Moreover, phylogenetic and quantitative real-time (qRT)-PCR analyses were completed to identify the key regulators of anthocyanin biosynthesis in Yan73 vegetative tissues. This study advances our understanding of anthocyanin biosynthesis in grapevine.

## Materials and Methods

### Plant Materials

Leaves, stems and tendrils were collected from *Vitis vinifera* Yan73 and Muscat Hamburg grapevines growing at Chateau Changyu Verna in Shaanxi, China (108°73 N, 34°33 E). Sample were collected following the methods described by Guan et al. (2012). Three biological replicates were collected for each tissue of each variety. When three to four new leaves emerged (01 May), 100 young fully expanded leaves of each replicate were collected from the third apical internode of grapevine shoot. At the same time as leaf sampling, the stems (nodes 1 through 3) and tendrils were collected. Each replicate was randomly collected from at least 60 whole grapevines on both sides of the canopy. All the samples were frozen in liquid nitrogen and stored at −80°C for metabolome and transcriptome analysis.

### Analysis of Grapevine Vegetative Tissue Anthocyanin Compounds

Anthocyanins were extracted from grapevine leaves, stems and tendrils and analyzed as previously described (Xie et al., 2018, 2019).

### RNA-Seq and Bioinformatics Analyses

Total RNA was extracted from grapevine leaves, stems and tendrils as previously described (Yang et al., 2018; Xie et al., 2019). For each grapevine tissue, a library was constructed and sequenced using the BGISEQ-500 platform (150 bp paired ends) (Beijing Genomic Institute^1^). A transcriptome analysis was conducted by mapping the clean reads to a reference *V. vinifera* genome^2^. Gene expression levels were estimated using the fragments per kilobase of exon per million fragments mapped (FPKM).

### Identification of R2R3-MYB Family Members in Grapevine Vegetative Tissues

Grapevine protein sequences were downloaded from a *V. vinifera* genome database^2^ to establish a local protein database. The Hidden Markov Model (HMM) profile of the R2R3-MYB domain (PF00249) was obtained from Pfam database^3^. This profile was used as a query to search the grapevine protein database using the HMMER program (version 3.0). Annotated protein sequences were examined for the presence of complete R2 and R3 domains with NCBI Batch Web CD-Search and HMM scan from the HMMER suite of Pfam. Finally, our RNA-seq data were selected to identify the differentially expressed R2R3-MYB family genes in the leaves, stems, and tendrils among Yan73 and its male parent.

### Phylogenetic Analysis

Phylogenetic analysis was performed according to our previous report (Xie et al., 2019). Multiple sequences were aligned with the MUSCLE algorithm-based AlignX module of MEGA5.1. Phylogenetic trees were constructed using the neighbor-joining method of MEGA5.1.

### qRT-PCR

A qRT-PCR assay was performed following our previous study (Xie et al., 2015). The primers used for the qRT-PCR are provided in Supplementary Table S1.

### Statistical Analysis

Histograms were prepared using OriginPro 8.5 (OriginLab Corporation).

## Results

### Similar Anthocyanin Profiles of Yan73 Grapevine Leaf, Stem, and Tendril Tissues

**FIGURE 2:**
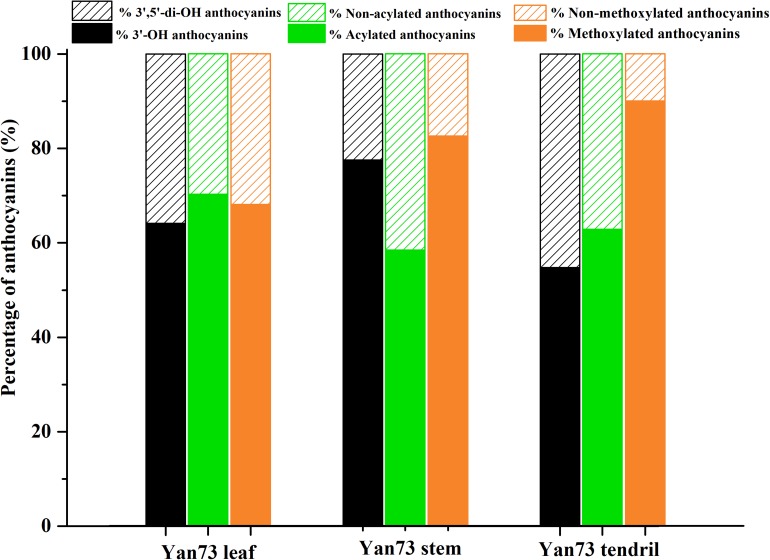
Proportions of different kinds of anthocyanins in Yan73 leaf, stem, and tendril tissues.

Anthocyanins in *V. vinifera* are mainly cyanidin-, delphinidin-, malvidin-, peonidin-, and petunidin-3-monoglucosides as well as the corresponding acetyl, *p*-coumaroyl, and caffeoyl derivatives (Guan et al., 2012; Rinaldo et al., 2015). In this study, the Yan73 grapevine red leaves contained 17 anthocyanins [four cyanidin (20.26%), two delphinidin (11.66%), four malvidin (19.24%), four peonidin (43.87%), and three petunidin (4.97%) derivatives], whereas the Yan73 red stems and tendrils both comprised seven anthocyanins (two cyanidin, two malvidin, and three peonidin derivatives) (Table 1). Delphinidin and petunidin derivatives were not detected in the Yan73 stems and tendrils. Additionally, the total anthocyanin concentration was more than 18-fold higher in Yan73 red leaves (2,441.04 mg/kg dry weight) than in Yan73 red stems (134.28 mg/kg dry weight) and tendrils (132.73 mg/kg dry weight) (Table 1). Although Yan73 leaves had more anthocyanins and a higher anthocyanin concentration than Yan73 stems and tendrils, all three tissues had similar anthocyanin profiles. Specifically, peonidin derivatives accounted for more than 40% of the total anthocyanins and peonidin-3-*O*-(*trans*-6-coumaryl)-glucoside was the most abundant anthocyanin in these grapevine vegetative tissues (Table 1). These findings were consistent with those of earlier analyses of the Yan73 leaf lamina (Guan et al., 2012). Furthermore, the percentages of 3′-OH, acylated, and methoxylated anthocyanins in Yan73 leaves, stems, and tendrils were higher than those of the corresponding 3′,5′-di-OH, non-acylated, and non-methoxylated anthocyanins (Figure 2). Similarly, Matus et al. (2017) determined that the Pinot Noir young leaves and tendrils and the Corvina Veronese buds tend to accumulate 3′-OH anthocyanins. The similar anthocyanin profiles in the Yan73 leaves, stems, and tendrils indicated that the anthocyanin biosynthesis in these vegetative tissues is regulated by similar mechanisms.

**TABLE 1:** Anthocyanin profiles of Yan73 grapevine leaf, stem, and tendril tissues.

Anthocyanin compounds	[M] + (m/z)	Concentration (mg/kg of dry weight)		
		Yan73 leaf	Yan73 stem	Yan73 tendril
**Cyanidin derivatives**				
Cyanidin-3-*O*-glucoside	449(287)	166.71 ± 0.27	10.78 ± 0.2	4.74 ± 0.18
Cyanidin-3-*O*-(6-acetyl)-glucoside	491(287,449)	17.71 ± 0.27	*ND*	*ND*
Cyanidin-3-*O*-(6-caffeoyl)-glucoside	611(287,449)	9.44 ± 0.22	*ND*	*ND*
Cyanidin-3-*O*-(6-coumaryl)-glucoside	595(449,287)	300.64 ± 17.16	12.61 ± 0.2	8.53 ± 0.27
Subtotal		494.49 ± 16.4	23.4 ± 0.4	13.27 ± 0.45
%		20.26	17.42	10.00
**Delphinidin derivatives**				
Delphinidin-3-*O*-glucoside	465(303)	105.47 ± 0.16	*ND*	*ND*
Delphinidin-3-*O*-(6-coumaryl)-glucoside	611(303,465)	179.21 ± 0.42	*ND*	*ND*
Subtotal		284.68 ± 0.27	0	0
%		11.66	0	0
**Malvidin derivatives**				
Malvidin-3-*O*-glucoside	493(331)	139.34 ± 6.45	15.64 ± 0.33	25.91 ± 0.18
Malvidin-3-*O*-(6-acetyl)-glucoside	535(331,493)	42.99 ± 1.32	*ND*	*ND*
Malvidin-3-*O*-(*cis*-6-coumaryl)-glucoside	639(493,331)	19.84 ± 0.07	*ND*	*ND*
Malvidin-3-*O*-(*trans*-6-coumaryl)-glucoside	639(493,331)	267.44 ± 0.18	14.52 ± 0.04	34.14 ± 0.40
Subtotal		469.6 ± 5.25	30.16 ± 0.29	60.05 ± 0.58
%		19.24	22.46	45.24
**Peonidin derivatives**				
Peonidin-3-*O*-glucoside	463(301)	263.33 ± 5.02	29.36 ± 0.07	18.67 ± 0.38
Peonidin-3-*O*-(6-caffeoyl)-glucoside	625(463,301)	42.4 ± 0.18	*ND*	*ND*
Peonidin-3-*O*-(*cis*-6-coumaryl)-glucoside	609(463,301)	68.52 ± 0.22	1.46 ± 0.76	0.94 ± 0.07
Peonidin-3-*O*-(*trans*-6-coumaryl)-glucoside	609(463,301)	696.72 ± 0.81	49.89 ± 0.02	39.8 ± 0.42
Subtotal		1070.97 ± 5.87	80.72 ± 0.80	59.41 ± 0.74
%		43.87	60.11	44.76
**Petunidin derivatives**				
Petunidin-3-*O*-glucoside	479(317)	51.94 ± 0.02	*ND*	*ND*
Petunidin-3-*O*-(*cis*-6-coumaryl)-glucoside	625(317,479)	0.66 ± 0.29	*ND*	*ND*
Petunidin-3-*O*-(*trans*-6-coumaryl)-glucoside	625(317,479)	68.69 ± 0.33	*ND*	*ND*
Subtotal		121.3 ± 0.07	0	0
%		4.97	0	0
Total		2441.04 ± 4.95	134.28 ± 0.91	132.73 ± 1.76

*Data are presented as the mean ± standard deviation of three biological replicates; ND, not detected.*

In contrast, no anthocyanins were detected in the Yan73 male parent (Muscat Hamburg) leaves, stems, and tendrils, implying that the anthocyanin accumulation in the Yan73 leaves, stems, and tendrils is attributed to a fully active anthocyanin biosynthesis pathway.

### Genes Related to Anthocyanin Biosynthesis in Yan73 Vegetative Tissues

**FIGURE 3:**
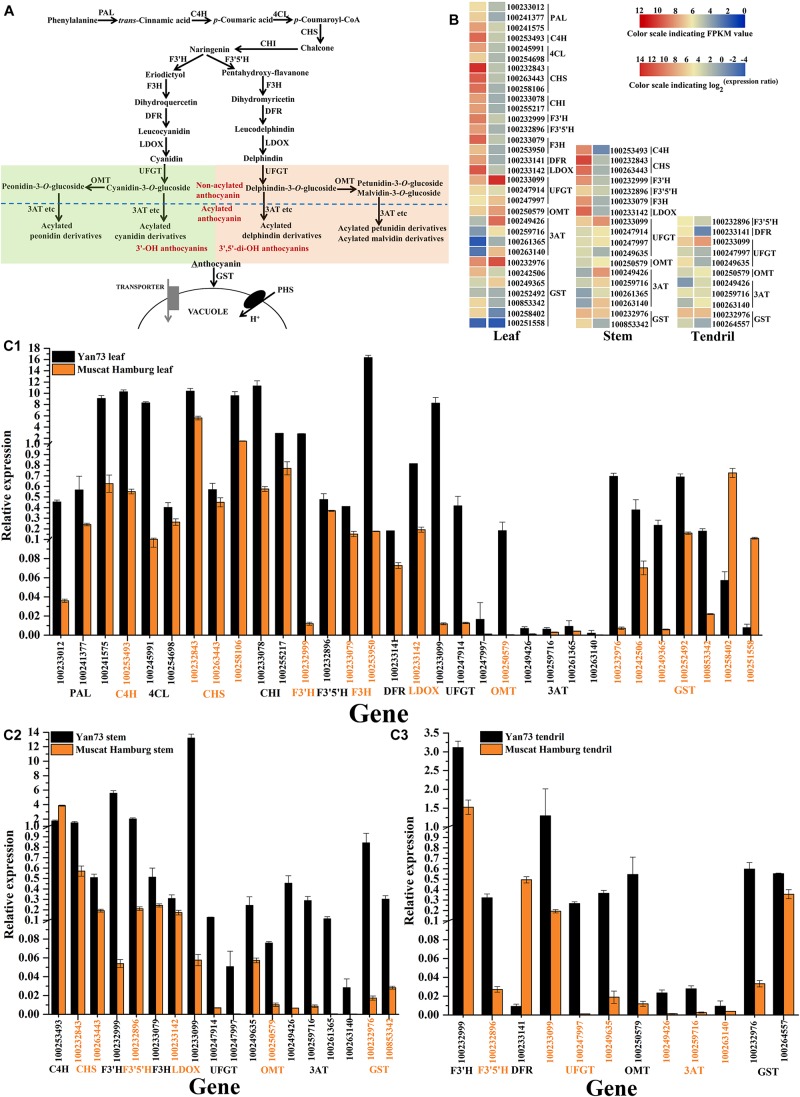
Expression analysis of anthocyanin biosynthesis genes in Yan73 and its male parent (Muscat Hamburg) leaf, stem and tendril tissues. **(A)** Schematic of anthocyanin biosynthesis pathways in grapevine vegetative tissues. PAL, phenylalanine ammonia lyase; C4H, *trans*-cinnamate 4-monooxygenase; 4CL, 4-coumarate: CoA ligase; CHS, chalcone synthase; CHI, chalcone isomerase; F3′H, flavonoid 3′-hydroxylase; F3′5′H, flavonoid 3′,5′-hydroxylase; F3H, flavanone 3-hydroxylase; DFR, dihydroflavonol 4-reductase; LDOX, leucoanthocyanidin dioxygenase; UFGT, anthocyanidin 3-*O*-glucosyltransferase; OMT, *O*-methyltransferase; 3AT, anthocyanin 3-*O*-glucoside-6′′-*O*-acyltransferase; GST, glutathione *S*-transferase. **(B)** RNA-seq analysis of anthocyanin biosynthesis genes in Yan73 and its male parent leaf, stem, and tendril tissues. Each gene expression pattern is presented on two grids: the left presents the FPKM values of Yan73 red leaf, stem and tendril tissues, whereas the right presents the log_2_^(Yan73 leaf,stem,tendril/Yan73 male parent leaf,stem,tendril)^ values. The grid in the above with six colors indicates the absolute expression levels in Yan73 leaf, stem and tendril, with the FPKM values 0–2^2^, 2^2^–2^4^, 2^4^–2^6^, 2^6^–2^8^, 2^8^–2^10^, and 2^10^–2^12^ represented by 0–2, 2–4, 4–6, 6–8, 8–10, and 10–12, respectively. **(C)** qRT-PCR analysis of anthocyanin biosynthesis genes in Yan73 and its male parent leaves **(C1)**, stems **(C2)**, and tendrils **(C3)**. The data were normalized against *VvUbiquitin* expression data. Error bars illustrate the standard deviations for three biological replicates.

To explore why the anthocyanin biosynthesis pathway (Figure 3A) is activated in Yan73 leaves, stems and tendrils, the transcriptomes of these three tissues were compared between Yan73 and its male parent. A total of 32 differentially expressed genes (DEGs) related to the anthocyanin biosynthesis pathway were identified in the leaves, whereas 18 and 11 DEGs were detected in the stems and tendrils, respectively (Figure 3B). With the exception of two *VvGST* isogenes (NCBI accession number 100258402 and 100251558), other 30 DEGs in leaves exhibited up-regulated expression in Yan73, including early biosynthesis genes [*VvPAL*(100233012, 100241377 and 100241575), *VvC4H*(100253493), *Vv4CL*(100245991and 100254698), *VvCHS*(100232843, 100263443, and 100258106), *VvCHI*(100233078 and 100255217), *VvF3′H*(100232999), *VvF3′5′H*(100232896), and *VvF3H*(100233079 and 100253950)], late biosynthesis genes [*VvDFR*(100233141) and *VvLDOX*(100233142)], anthocyanin modification genes [*VvUFGT*(100233099, 100247914, and 100247997), *VvOMT*(100250579) (Hugueney et al., 2009), and *Vv3AT*(100249426, 100259716, 100261365, and 100263140) (Rinaldo et al., 2015)], and an anthocyanin transport gene [*VvGST4*(100232976) (Conn et al., 2008)] (Figure 3B). Of the DEGs in the stems and tendrils, 17 and 10 were more highly expressed in Yan73 than in Yan73 male parent, respectively. Specifically, three types of anthocyanin modification genes (*VvUFGT*, *VvOMT*, and *Vv3AT*), and an anthocyanin transport gene (*VvGST4*) exhibited up-regulated expression in Yan73 stems and tendrils (Figure 3B). However, *VvC4H* (100253493) and *VvDFR* (100233141) expression levels were down-regulated in Yan73 stems and tendrils (Figure 3B), respectively. The F3′H and F3′5′H enzymes are important for controlling the production of 3′-OH anthocyanins and 3′,5′-di-OH anthocyanins in anthocyanin biosynthetic pathway (Figure 3A). In this study, *VvF3*′*H* and *VvF3*′*5*′*H* expression levels were up-regulated in Yan73 leaves and stems (Figure 3B), whereas in tendrils, *VvF3*′*H* expression levels were slightly higher in Yan73 (FPKM value of 169.85) than in Yan73 male parent (FPKM value of 116.32), but this difference was not significant. Additionally, *VvF3*′*H* was more highly expressed than *VvF3*′*5*′*H* in Yan73 leaves, stems, and tendrils (Figure 3B). These findings are confirmed by our qRT-PCR data (Figure 3C).

Consequently, the expression levels of 30, 17, and 10 anthocyanin biosynthesis genes were up-regulated in Yan73 leaves, stems, and tendrils, respectively, indicating anthocyanin biosynthesis in Yan73 vegetative tissues is regulated by transcription factors.

### R2R3-MYB Family Genes Responsible for Anthocyanin Biosynthesis in Grapevine Vegetative Tissues

**FIGURE 4:**
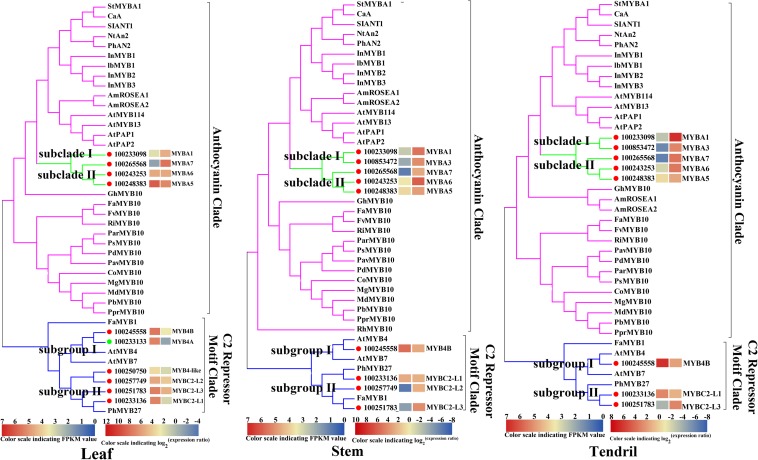
Phylogenetic relationships among R2R3-MYB family members detected in Yan73 and its male parent leaf, stem, and tendril tissues (based on RNA-seq data) and R2R3-MYBs from other plant species. The gene IDs are listed in Supplementary Tables S2–S5. The up- and down-regulated transcription factors in Yan73 red leaf, stem and tendril tissues are indicated with red and green dots, respectively, and the corresponding transcript patterns are presented on two grids: the left presents the FPKM values of Yan73 leaves, stems, and tendrils, whereas the right presents the log_2_^(Yan73 leaf,stem,tendril/Yan73 male parent leaf,stem,tendril)^ values. The grid on the left with seven colors indicates the absolute expression levels in Yan73 red leave, stem and tendril tissues, with the FPKM values 0-2^1^, 2^1^-2^2^, 2^2^-2^3^, 2^3^-2^4^, 2^4^-2^5^, 2^5^-2^6^, and 2^6^-2^7^ represented by 0-1, 1-2, 2-3, 3-4, 4-5, 5–6, and 6-7, respectively. Each functional clade is highlight.

**FIGURE 5:**
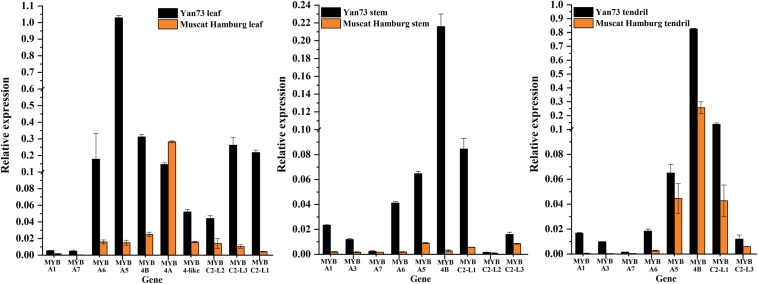
Relative expression levels of transcription factor genes associated with anthocyanin biosynthesis in Yan73 and its male parent (Muscat Hamburg) leaf, stem, and tendril tissues. The data are derived from a qRT-PCR analysis and are normalized against *VvUbiquitin* expression data. Error bars illustrate the standard deviations for three biological replicates.

To identify the key regulators inducing the expression of a set of anthocyanin genes in Yan73 leaves, stems, and tendrils, we screened our RNA-seq data for R2R3-MYB family members. A total of 65, 69, and 50 DEGs encoding R2R3-MYB proteins were identified in the leaves (Supplementary Table S2), stems (Supplementary Table S3), and tendrils (Supplementary Table S4), respectively, of which the expression levels of 47, 31, and 31 genes were up-regulated in Yan73 leaves, stems, and tendrils, respectively. Phylogenetic trees comprising these differentially expressed R2R3-MYB genes in the leaves (Supplementary Figure S1), stems (Supplementary Figure S2), and tendrils (Supplementary Figure S3) as well as the genes encoding known MYB anthocyanin regulators from other species were constructed to identify candidate R2R3-MYB transcription factors putatively involved in the anthocyanin biosynthesis in Yan73 leaves, stems, and tendrils. Structural homology among the MYB proteins from various plant species may indicate the pathways they regulate are generally similar, as are their effects (activation or repression) on the pathways (Aharoni et al., 2001). In our phylogenetic trees, anthocyanin-related MYB transcription factors from various plant species were clustered in the same anthocyanin clade (Figure 4), implying these MYBs might have similar functions in regulating anthocyanin biosynthesis. On the basis of our RNA-seq results for grapevine leaves, stems, and tendrils, two groups of R2R3-MYB genes (Subclades I and II) were identified in the anthocyanin clade. Furthermore, the expression levels of *VvMYBA1*(100233098) in Subclade I and *VvMYBA5*(100248383), *VvMYBA6*(100243253), and *VvMYBA7*(100265568) in Subclade II were up-regulated in Yan73 leaves, stems, and tendrils, suggesting both groups of genes have a role in the anthocyanin accumulation of these Yan73 vegetative tissues. Among both groups of *VvMYBA* genes, *VvMYBA5* is the most highly expressed in Yan73 leaves, stems, and tendrils, with FPKM values of 63.15, 13.446, and 12.52, respectively, followed by *VvMYBA6* (Supplementary Tables S2–S4), suggesting VvMYBA5 and VvMYBA6 maybe the predominant regulators of vegetative anthocyanins in Yan73. We did not detect *VvMYBA3*(100853472) expression in Yan73 leaves, but it was up-regulated in Yan73 stems and tendrils. These results are confirmed by our qRT-PCR data (Figure 5).

The expression of anthocyanin-related genes is regulated not only by transcriptional activators but also by repressors (Aharoni et al., 2001). In grapevine, Cavallini et al. (2015) reported two groups of R2R3-MYB C2 repressors that distinctly regulate different branches of the phenylpropanoid pathway. Specifically, Subgroup I genes repress the biosynthesis of phenolic compounds, whereas Subgroup II genes negatively regulate anthocyanin and proanthocyanidin biosynthesis. In this study, the expression levels of the Subgroup II genes were up-regulated in Yan73 red leaves, stems, and tendrils, especially *VvMYBC2-L1*(100233136) and *VvMYBC2-L3*(100251783) (Figure 4). These results indicate this group of R2R3-MYB C2 repressors may play a role in anthocyanin accumulation of Yan73 leaf, stem, and tendril. However, the expression of *VvMYB4A*(100233133) from Subgroup I was down-regulated in Yan73 leaves, although the expression of *VvMYB4B*(100245558) from Subgroup I was up-regulated in Yan73 leaves, stems, and tendrils. These results are confirmed by our qRT-PCR data (Figure 5).

## Discussion

### Anthocyanin Profiles Differ Among Yan73 Vegetative and Reproductive Tissues

Yan73 grapevine leaf, stem and tendril showed similar anthocyanin profile, indicating that anthocyanin biosynthesis in Yan73 vegetative tissues is regulated by similar mechanisms. However, the anthocyanin profiles of the three analyzed Yan73 vegetative tissues differed from those of Yan73 reproductive tissues, such as the berry skin and flesh. Malvidin-3-*O*-glucoside and peonidin-3-*O*-glucoside are reportedly the most abundant anthocyanins in Yan73 berry skin and flesh, respectively (Guan et al., 2012), whereas the most abundant anthocyanin in Yan73 leaves, stems, and tendrils is peonidin-3-*O*-(*trans*-6-coumaryl)-glucoside (Table 1). Furthermore, we previously revealed that 3′,5′-di-OH and non-acylated anthocyanins are the predominant anthocyanins in Yan73 berry skin and that Yan73 berry flesh has similar concentrations of 3′-OH and 3′,5′-di-OH anthocyanins and higher concentrations of non-acylated anthocyanins (Xie et al., 2015, 2018), whereas Yan73 vegetative tissues had higher percentages of 3′-OH and acylated anthocyanins. These differences in the anthocyanin profiles of Yan73 vegetative and reproductive tissues suggest that the regulatory mechanisms underlying the anthocyanin biosynthesis in these vegetative tissues may differ.

In contrast, no anthocyanins were detected in the vegetative tissues of Yan73 male parent, suggesting anthocyanin biosynthesis in the Yan73 vegetative tissues is attributed to a fully active anthocyanin biosynthesis pathway.

### Anthocyanin Biosynthesis Genes Are Activated in Yan73 Vegetative Tissues

A total of 32 DEGs related to the anthocyanin biosynthesis pathway were identified in the leaves, whereas 18 and 11 DEGs were detected in the stems and tendrils, respectively (Figure 3B). This was consistent with the observed greater number of anthocyanins and higher anthocyanin concentration in Yan73 leaves than in the stems and tendrils (Table 1). Among these DEGs, the expression levels of 30, 17, and 10 anthocyanin biosynthesis genes were up-regulated in Yan73 leaves, stems, and tendrils, respectively (Figure 3B). Only Two *VvGST* isogenes, *VvC4H* and *VvDFR* expression levels were down-regulated in Yan73 leaves, stems and tendrils (Figure 3B), respectively, indicating slight differences in the activities of anthocyanin pathways among Yan73 these tissues. In fact, *C4H* and *DFR* genes do not specifically regulate anthocyanin biosynthesis, but they are also involved in the production of other phenylpropanoid compounds, such as flavanols, flavonols (Xie et al., 2019), hydroxycinnamic acids (Cavallini et al., 2015), and stilbenes (Holl et al., 2013).

The F3′H and F3′5′H enzymes are important for controlling the production of 3′-OH anthocyanins and 3′,5′-di-OH anthocyanins (Figure 3A). In this study, *VvF3*′*H* was more highly expressed than *VvF3*′*5*′*H* in Yan73 vegetative tissues (Figure 3B), which is consistent with the observed higher 3′-OH anthocyanin concentration than 3′,5′-di-OH anthocyanin concentration in Yan73 vegetative tissues. The final anthocyanin modification steps in the grapevine anthocyanin biosynthesis pathway include the glycosylation by UFGT, methylation by OMT, and acylation by 3AT (Figure 3A). In this study, the expression levels of *VvUFGT*, *VvOMT*, and *Vv3AT* were up-regulated in the Yan73 vegetative tissues (Figure 3B), which explains the higher percentages of acylated and methoxylated anthocyanins than those of the corresponding non-acylated, and non-methoxylated anthocyanins in Yan73 these tissues (Figure 2). Earlier investigations revealed that *VvUFGT* (Kobayashi et al., 2001), *VvOMT* (Hugueney et al., 2009), and *Vv3AT* (Conn et al., 2008) are critical for anthocyanin biosynthesis in grape berry. In our study, these three types of genes are also important for the anthocyanin biosynthesis in Yan73 vegetative tissues. Of five grapevine GST genes analyzed by Conn et al. (2008), only *VvGST1* and *VvGST4* are involved in anthocyanin transport. In the current study, we did not detect *VvGST1* expression, but the *VvGST4* (100232976) expression was strongly up-regulated in Yan73 vegetative tissues (Figure 3B), suggesting *VvGST4* gene may control the anthocyanin transport of Yan73 these vegetative tissues. To the best of our knowledge, there has been relatively little research on the anthocyanin biosynthesis in the teinturier grapevine vegetative tissues. Only Jeong et al. (2006) reported that *VvUFGT* expression was detected in the vegetative tissues of a teinturier grapevine cultivar (Bailey Alicant A) and might be responsible for anthocyanin biosynthesis of Bailey Alicant A vegetative tissues.

Consequently, the expression levels of 30, 17, and 10 anthocyanin biosynthesis genes were up-regulated in Yan73 leaves, stems, and tendrils, respectively. Especially, the anthocyanin-specific genes *VvUFGT*, *VvOMT*, *Vv3AT*, and *VvGST4* exhibited up-regulated expression in these Yan73 vegetative tissues. These results imply that anthocyanin biosynthesis in Yan73 vegetative tissues is regulated by transcription factors. Transcription factors, as different from most proteins encoded by structural genes, tend to control multiple pathway steps. Moreover, transcription factor families, including MYB, bHLH, WD40, WRKY, and NAC have been reported to coordinately regulate the expression of a series of anthocyanin biosynthesis genes to control anthocyanin production (Zhou et al., 2015; Amato et al., 2016; Xie et al., 2019).

### Transcriptional Activators and Repressors Coordinately Regulate Anthocyanin Biosynthesis in Grapevine Vegetative Tissues

Recently, extensive research has confirmed that the expression of anthocyanin biosynthesis genes is controlled by the MBW protein complex, with R2R3-MYB transcription factors playing a central role in the coordinated activation of genes specific to anthocyanin pathways (Albert et al., 2014; Costantini et al., 2015). The expression levels of *VvMYBA1* in Subclade I and *VvMYBA5*, *VvMYBA6*, and *VvMYBA7* in Subclade II were up-regulated in Yan73 vegetative tissues (Figure 4), suggesting both groups of genes have a role in the anthocyanin accumulation of these Yan73 vegetative tissues. Earlier investigations demonstrated that *MYBA1* and *MYBA2* on chromosome 2 determined anthocyanin biosynthesis in grape berry (Walker et al., 2007), whereas *MYBA5*, *MYBA6*, and *MYBA7* on chromosome 14 control the anthocyanin biosynthesis in grapevine vegetative tissues (Matus et al., 2017). However, Matus et al. (2017) claimed that although *MYBA5*, *MYBA6*, and *MYBA7* regulate anthocyanin accumulation of grapevine vegetative tissues, it is possible that in particular cases, the anthocyanin biosynthesis of the grapevine vegetative tissues may also be mediated by *MYBA1*. This possibility is supported by the fact *MYBA1* is also expressed in some vegetative tissues able to accumulate anthocyanins, such as the leaves of a teinturier cultivar (Jeong et al., 2006), GLRaV-infected leaves (Gutha et al., 2010), and senescing leaves (Matus et al., 2017). Our study reveals that *VvMYBA1* along with *VvMYBA5*, *VvMYBA6*, and *VvMYBA7* coordinately regulate anthocyanin biosynthesis in Yan73 vegetative tissues. *VvMYBA5*, *VvMYBA6*, and *VvMYBA7* are reportedly insufficient for promoting the accumulation of 3′,5′-di-OH anthocyanins because they are incapable of activating the expression of *F3*′*5*′*H* genes. However, *MYBA1* can induce *F3*′*5*′*H* expression and the corresponding accumulation of 3′,5′-di-OH anthocyanins (Matus et al., 2017). Our metabolic data revealed that 3′,5′-di-OH anthocyanins are highly abundant in Yan73 vegetative tissues, although not as abundant as 3′-OH anthocyanins (Figure 2). This further support that VvMYBA1 participate in the regulation of anthocyanin biosynthesis in Yan73 vegetative tissues. Additionally, among both groups of *VvMYBA* genes, *VvMYBA5* is the most highly expressed in Yan73 vegetative tissues, followed by *VvMYBA6*, indicating VvMYBA5 and VvMYBA6 maybe the predominant regulators of vegetative anthocyanins in Yan73. This further explain the relatively high 3′-OH anthocyanin concentrations in the Yan73 vegetative tissues (Figure 2). *VvMYBA3* expression was not detected in Yan73 leaves, but it was up-regulated in Yan73 stems and tendrils (Figure 4). The VvMYBA3 was previously reported to be non-functional in berry anthocyanin accumulation, likely because of the steric competition between functional VvMYBA1 and VvMYBA2 (Fournier-Level et al., 2009). In our study, the *VvMYBA3* expression level was lower than that of *VvMYBA1* in Yan73 stems and tendrils, implying that *VvMYBA3* may be less involved in anthocyanin biosynthesis of Yan73 vegetative tissue.

Anthocyanin-related gene expression is regulated by transcriptional activators as well as repressors (Aharoni et al., 2001). Previous studies identified many R2R3-MYB transcriptional repressors, including PpMYB18 in peach (Zhou et al., 2019), FaMYB1 and FcMYB1 in strawberry (Aharoni et al., 2001; Salvatierra et al., 2013), PtMYB182 in poplar (Yoshida et al., 2015), and PhMYB27 in petunia (Albert et al., 2011), that function coordinately with transcriptional activators to fine-tune anthocyanin biosynthesis during plant development. In this study, the expression levels of the Subgroup II genes were up-regulated in Yan73 vegetative tissues, especially VvMYBC2-L1 and VvMYBC2-L3 (Figure 4), indicating this group of R2R3-MYB C2 repressors may play a role in anthocyanin accumulation of Yan73 vegetative tissues. Although Subgroup II genes were identified as transcriptional repressors, they are highly expressed during the active stages of anthocyanin accumulation in Yan73 vegetative tissues. In fact, until now, considering the relationship between the expression of R2R3-MYB repressor genes and anthocyanin accumulation, two expression pattern types have been proposed (Chen et al., 2019). The expression of one class of R2R3-MYB repressor genes, including apple *MdMYB16* (Xu et al., 2017), poplar *MYB182*(Yoshida et al., 2015), and *Ginkgo biloba GbMYBF2* (Xu et al., 2014), is negatively associated with anthocyanin accumulation. The expression of these genes prevents the ectopic accumulation of anthocyanins. The other class of repressor genes, including strawberry *FaMYB1* (Aharoni et al., 2001), peach *PpMYB18* (Zhou et al., 2019), and petunia *MYB27* (Albert et al., 2014), are highly expressed during the active stages of anthocyanin accumulation to provide feedback repression to finely control and limit anthocyanin levels. In the current study, the R2R3-MYB C2 repressor genes of Subgroup II belong to the second gene class and are highly expressed during the active stages of anthocyanin accumulation in Yan73 vegetative tissues. To explain this expression pattern, the following negative feedback loop regulating anthocyanin biosynthesis was proposed: activators activate repressors, repressors repress activators, and repressors repress repressors (Albert et al., 2014; Zhou et al., 2019). In this study, the up-regulated expression of two groups of VvMYBA transcription activator genes induced anthocyanin accumulation and may have also activated the expression of a set of R2R3-MYB C2 repressor genes to maintain suitable anthocyanin accumulation in Yan73 vegetative tissues. However, the expression pattern behaviors of Subgroup I genes in Yan73 leaves, stems, and tendrils were not consistent. The expression of *VvMYB4B* from Subgroup I was up-regulated in Yan73 leaves, stems, and tendrils, whereas the expression of *VvMYB4A* from Subgroup I was down-regulated in Yan73 leaves. Therefore, Subgroup I genes were not well related to anthocyanin accumulation in Yan73 vegetative tissues. In fact, to date, only Subgroup II genes, such as *MYB4-like* (Perez-Diaz et al., 2016), *MYBC2-L1* (Cavallini et al., 2015), *MYBC2-L2* (Zhu et al., 2019), and *MYBC2-L3* (Munoz et al., 2019), have been confirmed to participate in anthocyanin biosynthesis.

Our transcriptomic and metabolic data indicated that the up-regulated expression of two groups of VvMYBA transcription activator genes, including *VvMYBA1* on chromosome 2 and *VvMYBA5*, *VvMYBA6*, and *VvMYBA7* on chromosome 14, are responsible for the specific anthocyanin accumulation pattern in Yan73 vegetative tissues, although VvMYBA5 and VvMYBA6 maybe the predominant regulator of vegetative anthocyanins in Yan73. Simultaneously, the expression of a set of R2R3-MYB C2 repressor genes, mainly including *VvMYBC2-L1* and *VvMYBC2-L3*, is activated and involved in the negative regulation of anthocyanin biosynthesis in Yan73 vegetative tissues, thereby maintaining appropriate anthocyanin contents. However, future studies will need to determine why these R2R3-MYB transcriptional regulators are activated in Yan73 vegetative tissues.

## Conclusion

Yan73 grapevine leaf, stem and tendril showed similar anthocyanin profile, with relatively high percentages of 3′-OH, acylated and methoxylated anthocyanins in these Yan73 red vegetative tissues. Furthermore, peonidin-3-*O*-(*trans*-6-coumaryl)-glucoside was the most abundant anthocyanin in Yan73 red vegetative tissues. Our RNA-seq data revealed that a total of 30,17 and 10 anthocyanin biosynthesis genes exhibited up-regulated expression in Yan73 leaf, stem and tendril, respectively. Especially, the expression of anthocyanin-specific genes (*VvUFGT*, *VvOMT*, *Vv3AT*, and *VvGST4*) were all up-regulated in Yan73 vegetative tissues. These results indicate that anthocyanin biosynthesis in Yan73 vegetative tissues is regulated by transcription factors. Our transcriptomic and metabolic data revealed that the up-regulated expression of two groups of VvMYBA transcription activator genes, including *VvMYBA1* on chromosome 2 and *VvMYBA5*, *VvMYBA6*, and *VvMYBA7* on chromosome 14, are responsible for the specific anthocyanin patterns of Yan73 vegetative tissues, with *VvMYBA5* and *VvMYBA6* likely encoding the major regulators of the anthocyanin biosynthesis in Yan73 vegetative tissues. Simultaneously, the expression of a set of R2R3-MYB C2 repressor genes, mainly including *VvMYBC2-L1* and *VvMYBC2-L3*, is activated and may negatively regulate anthocyanin biosynthesis in Yan73 vegetative tissues, thereby maintaining suitable anthocyanin levels. The data presented herein contribute to our understanding of anthocyanin biosynthesis in grapevine.

## Data Availability Statement

The datasets generated for this study can be found in the NCBI SRA accession PRJNA610705.

## Author Contributions

SX designed the experiments, performed most of experiments, analyzed the data, and wrote the manuscript. ZZ provided all of the financial support and critical intellectual input into the study design. YL, HWC, JL, and HZC assisted in experiments and discussed the results.

## Conflict of Interest

The authors declare that the research was conducted in the absence of any commercial or financial relationships that could be construed as a potential conflict of interest.

## Footnotes

1 www.genomics.org.cn

2 https://www.ncbi.nlm.nih.gov/genome/?term=vitis+vinifera

3 http://pfam.xfam.org/
