# Supplementary material for: R2R3-MYB Transcription Factors Regulate Anthocyanin Biosynthesis in Grapevine Vegetative Tissues
Source: Front Plant Sci. 2020 May 7;11:527. doi: 10.3389/fpls.2020.00527 (PMC7221203; doi:10.3389/fpls.2020.00527)
Supplement: Supplementary file 1 [file Data_Sheet_1.pdf]

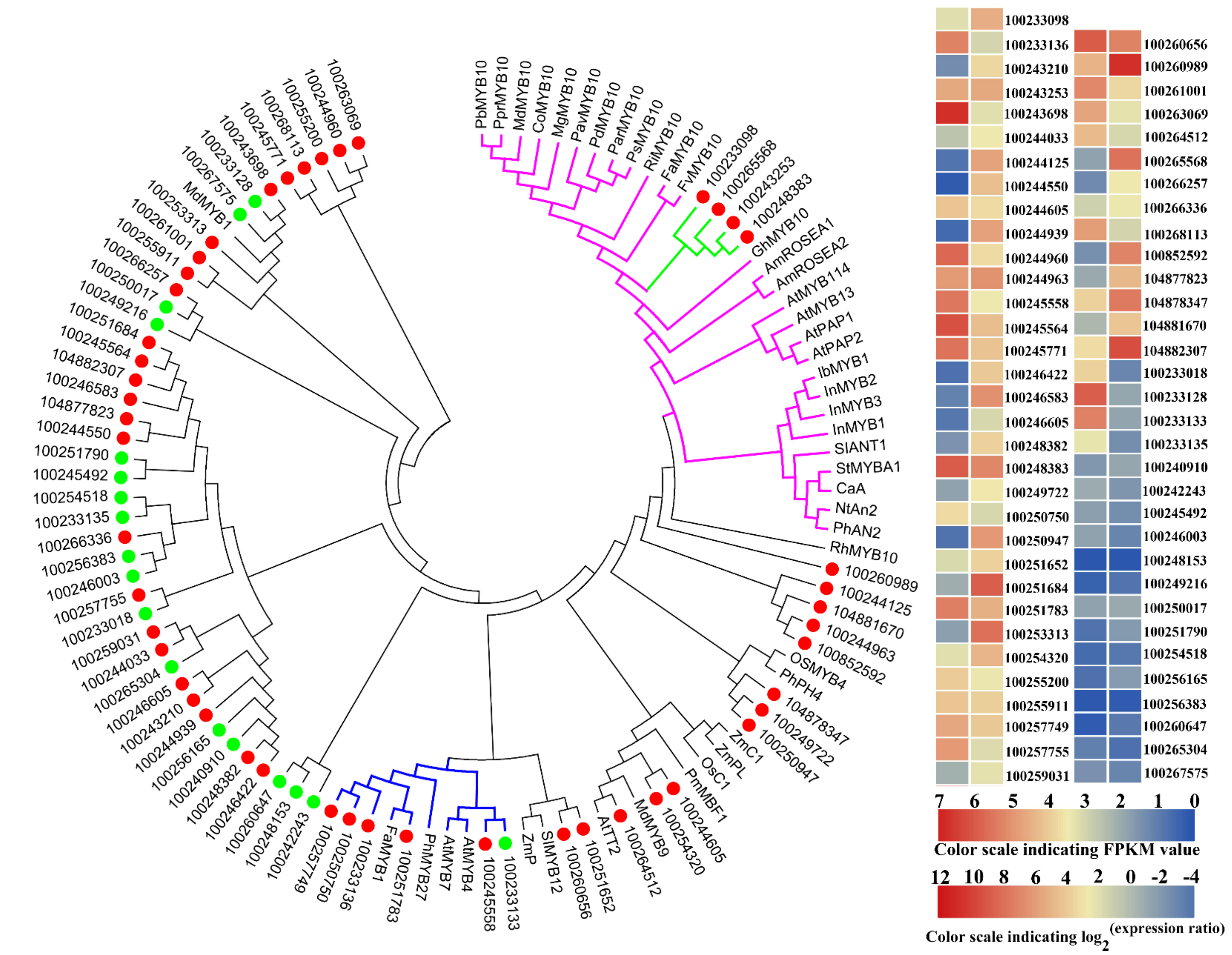

Supplementary Figure S1. Phylogenetic relationships among R2R3-MYB family members detected in Yan73 and its male parent leaves (based on RNA-seq data) and the R2R3-MYBs from other species. The gene IDs are listed in Supplementary Tables S2 and Tables S5. The up- and down-regulated transcription factors in Yan73 red leaf are indicated with red and green dots, respectively, and the corresponding transcript patterns are presented on the following two grids: the left presents the FPKM values of Yan73 red leaf, whereas the right presents the $\log_2^{(\text{Yan73 red leaf/ its male parent leaf})}$ values. The grid in the above with seven colors indicates the absolute expression levels in Yan73 leaf, with the FPKM values $0–2^1$, $2^1–2^2$, $2^2–2^3$, $2^3–2^4$, $2^4–2^5$, $2^5–2^6$ and $2^6–2^7$ represented by 0–1, 1–2, 2–3, 3–4, 4–5, 5–6 and 6–7 respectively. Each functional clade is highlighted.

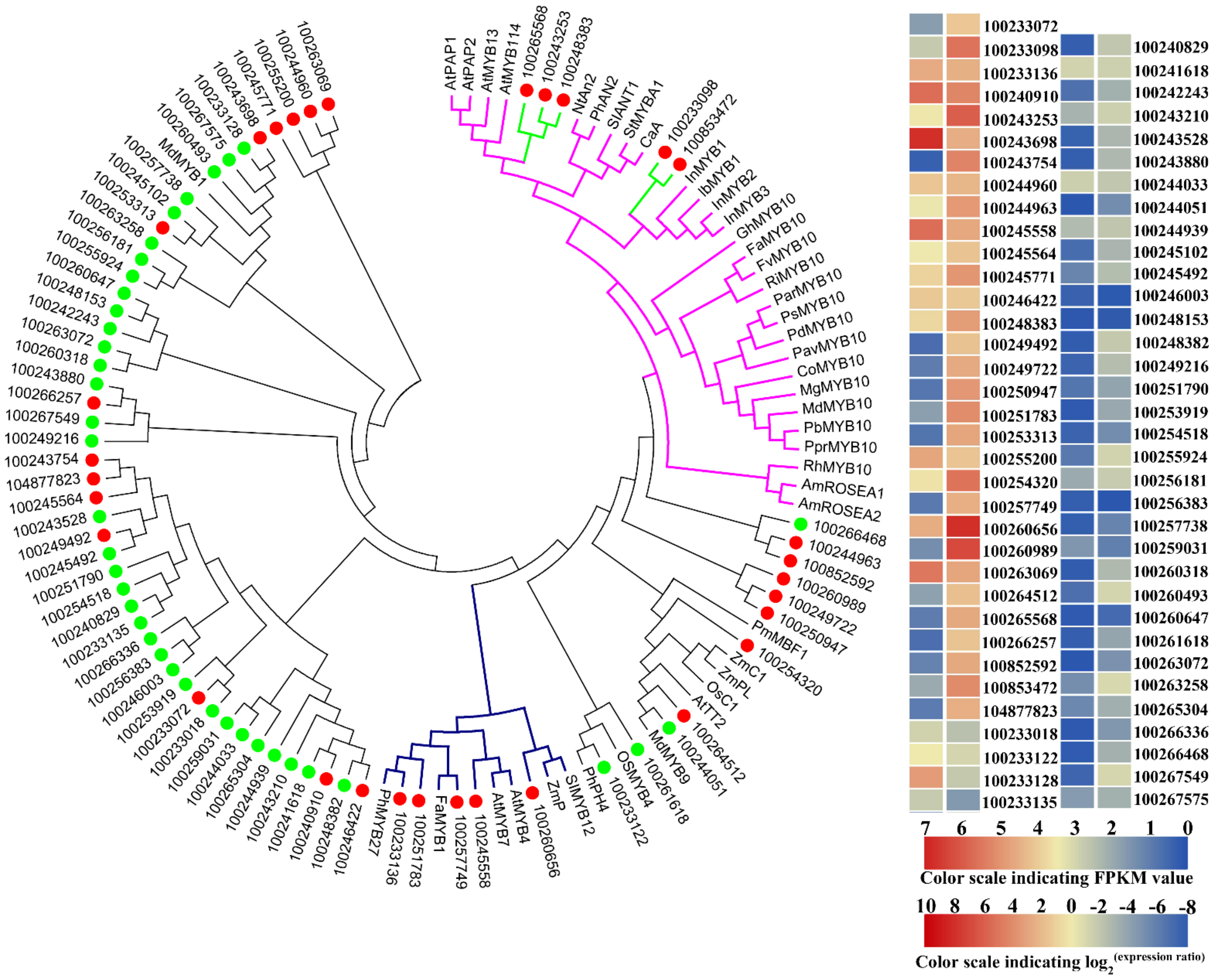

Supplementary Figure S2. Phylogenetic relationships among R2R3-MYB family members detected in Yan73 and its male parent stems (based on RNA-seq data) and the R2R3-MYBs from other species. The gene IDs are listed in Supplementary Tables S3 and Tables S5. The up- and down-regulated transcription factors in Yan73 red stem are indicated with red and green dots, respectively, and the corresponding transcript patterns are presented on the following two grids: the left presents the FPKM values of Yan73 red stem, whereas the right presents the $\log_2^{(\text{Yan73 red stem/ its male parent stem})}$ values. The grid in the above with seven colors indicates the absolute expression levels in Yan73 stem, with the FPKM values $0–2^1$, $2^1–2^2$, $2^2–2^3$, $2^3–2^4$, $2^4–2^5$, $2^5–2^6$ and $2^6–2^7$ represented by 0–1, 1–2, 2–3, 3–4, 4–5, 5–6 and 6–7 respectively. Each functional clade is highlighted.

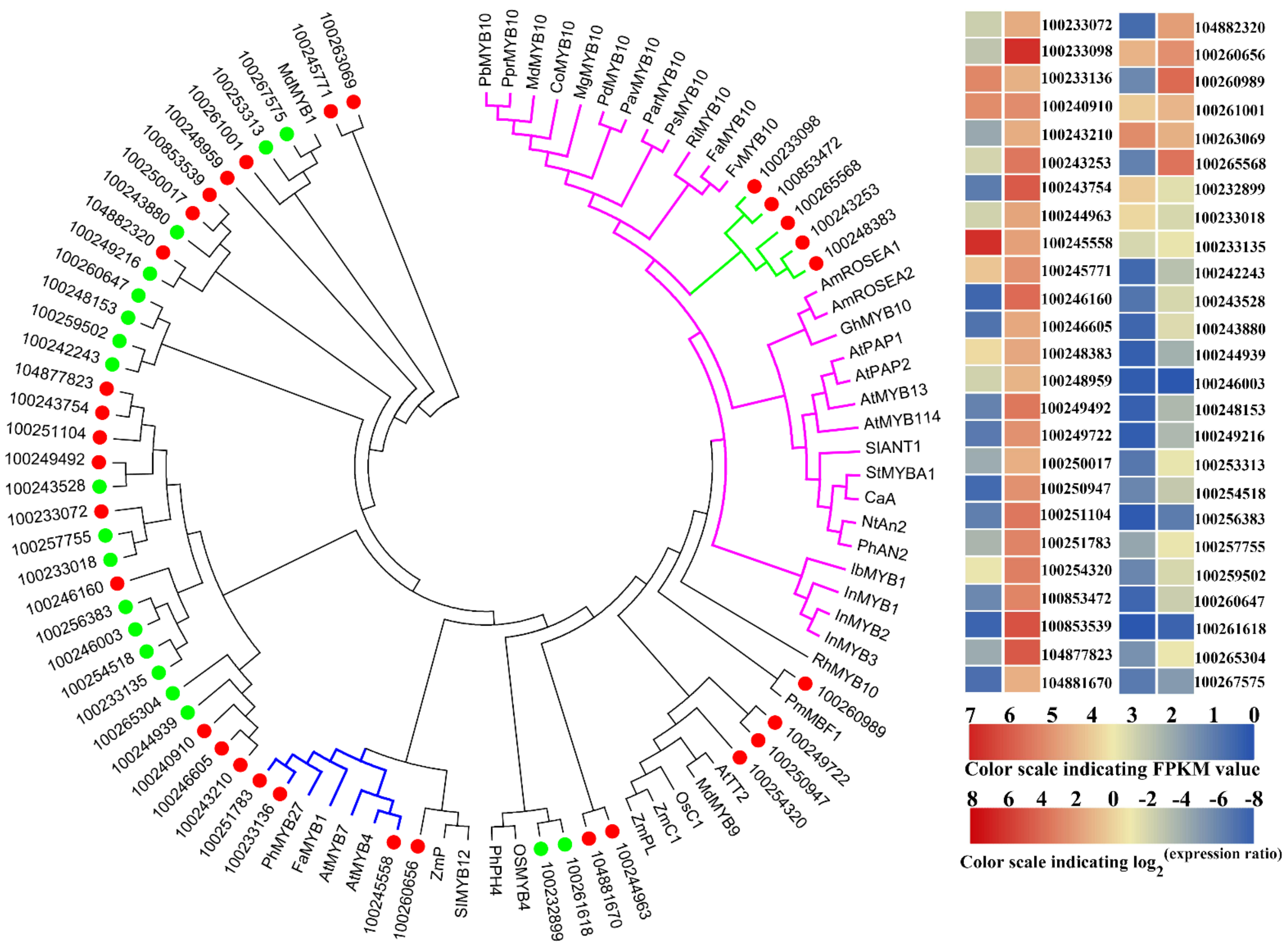

Supplementary Figure S3. Phylogenetic relationships among R2R3-MYB family members detected in Yan73 and its male parent tendrils (based on RNA-seq data) and the R2R3-MYBs from other species. The gene IDs are listed in Supplementary Tables S4 and Tables S5. The up- and down-regulated transcription factors in Yan73 red tendril are indicated with red and green dots, respectively, and the corresponding transcript patterns are presented on the following two grids: the left presents the FPKM values of Yan73 red tendril, whereas the right presents the $\log_2^{(\text{Yan73 red tendril /its male parent tendril})}$ values. The grid in the above with seven colors indicates the absolute expression levels in Yan73 tendril, with the FPKM values $0–2^1$, $2^1–2^2$, $2^2–2^3$, $2^3–2^4$, $2^4–2^5$, $2^5–2^6$ and $2^6–2^7$ represented by 0–1, 1–2, 2–3, 3–4, 4–5, 5–6 and 6–7 respectively. Each functional clade is highlighted.
